# Development of a self-management intervention to improve tamoxifen adherence in breast cancer survivors using an Intervention Mapping framework

**DOI:** 10.1007/s00520-020-05850-x

**Published:** 2020-10-29

**Authors:** Zoe Moon, Rona Moss-Morris, Myra S. Hunter, Lyndsay D. Hughes

**Affiliations:** Health Psychology Section, Institute of Psychiatry, Psychology & Neuroscience (IoPPN), King’s College London, 5th Floor Bermondsey Wing, London, SE1 9RT UK

**Keywords:** Adherence, Intervention Mapping, Breast cancer, Tamoxifen

## Abstract

**Objective:**

Up to 50% of women prescribed tamoxifen do not take it as prescribed for the full duration, which increases risk of recurrence and mortality. The current paper describes the development of a self-management intervention aiming to improve adherence in breast cancer survivors taking tamoxifen.

**Methods:**

The intervention was developed following an Intervention Mapping approach. The content of the intervention was determined by theories of health behaviour and empirical evidence. Development was an iterative process involving input from expert researchers, clinicians and patient representatives.

**Results:**

The intervention was designed to improve both intentional and unintentional non-adherence. Key features included modifying unhelpful illness and treatment beliefs, improving confidence for coping with side effects and developing strategies for remembering to take tamoxifen.

**Conclusion:**

Intervention Mapping proved a useful tool for developing an intervention which is grounded in theory and empirical evidence. The intervention has the potential to improve adherence in breast cancer survivors but needs to be trialled before the effectiveness of the intervention can be determined.

**Supplementary Information:**

The online version contains supplementary material available at 10.1007/s00520-020-05850-x.

## Introduction

Women with oestrogen receptor positive early breast cancer are prescribed hormone therapy (HT) such as tamoxifen for up to 10 years post primary treatment in order to reduce the risk of cancer recurrence. Studies show that taking tamoxifen for up to 10 years can reduce the risk of cancer recurrence by 50% [1].

However, by the fifth year of treatment, up to 50% of women have stopped taking their tamoxifen, known as non-persistence, or are not taking it as prescribed, known as non-adherence [2, 3]. Both non-adherence and non-persistence are associated with increased risk of recurrence and mortality [4, 5]. Improving adherence in this population has the potential to further improve long-term survival. However, whilst there is increasing interest in identifying predictors or correlates of HT non-adherence, few studies have used the results to develop interventions, despite this being identified as a research priority [6]. This paper describes the development of an evidence-based and theory-driven self-management intervention to support women taking tamoxifen and improve adherence rates.

Across illnesses and medications, interventions to improve adherence tend to have little success [7]. A number of studies have shown that providing educational materials to patients prescribed aromatase inhibitors (AIs), a similar class of drugs to tamoxifen, does not improve adherence rates [8]. However, there are several issues with the previous literature which may contribute to this lack of treatment efficacy. Firstly, the majority of previous interventions have not considered both intentional and unintentional non-adherence, a factor which may reduce effectiveness of adherence interventions. Intentional non-adherence occurs when a patient makes a deliberate decision not to take medication as prescribed, whereas unintentional non-adherence occurs when a patient may forget to take their medication or they do not understand the instructions. Previous research has demonstrated a distinction between factors which contribute to intentional and unintentional non-adherence, confirming the need to target these factors independently [9].

Secondly, many adherence interventions do not have a theoretical basis. Grounding interventions in theory is likely to improve their effectiveness by identifying theoretical constructs on which to focus the intervention, specifying effective behaviour change methods to address these constructs and allowing evaluation of why improvements may have occurred [10]. This paper describes the development of an intervention to address tamoxifen non-adherence targeted at women with sub-optimal levels of adherence. Intervention development was based on two models of health behaviour: the Common Sense Model of Illness Representations (CSM) and the Theory of Planned Behaviour (TPB). The CSM assumes that people are active problem solvers who will try and make sense of and reduce a given illness or health threat. The model suggests that whether someone engages in a health behaviour such as treatment adherence depends on how well that health behaviour fits with their personal representation of their illness [11]. The TPB proposes that whether or not someone takes their medication will depend on their intentions to take it, others’ attitudes towards taking it (subjective norms) and their beliefs about their ability to take the medication (perceived behavioural control) [12]. Both of these models have been shown to explain non-adherence to tamoxifen [9].

### Framework for intervention development

The intervention was developed in accordance with Medical Research Council (MRC) guidance for the development of complex interventions, which recommends that preliminary development work should be carried out prior to the commencement of any large RCT and that interventions should be based on theory and empirical evidence to allow for replication and wide adoption of successful techniques [13]. Intervention Mapping (IM) was used as a framework for developing the intervention, as it provides a clear protocol for ensuring that interventions are grounded in theory [14]. Intervention development has been described following recent guidance to improve transparency and enhance quality of intervention development and practice [15].

## Method

**Fig. 1:**
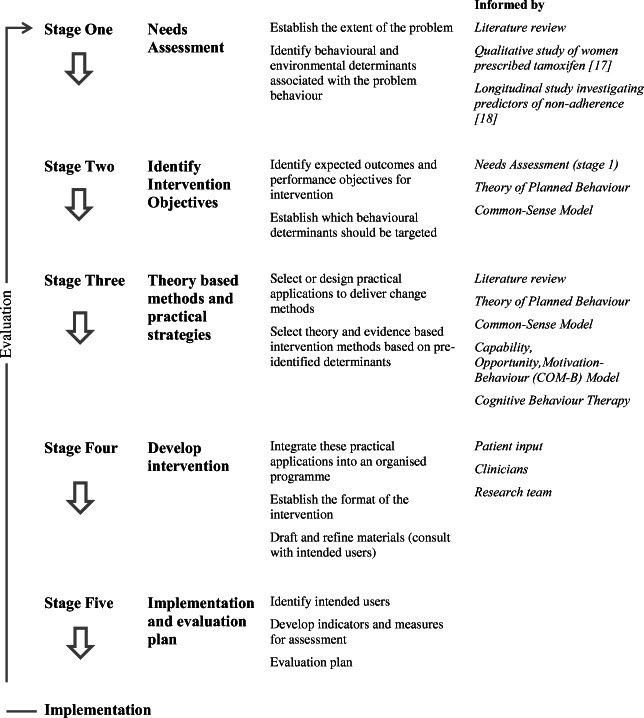
Adapted interventon mapping framework

The IM framework involves a five-stage process: (1) needs assessment, (2) identifying intervention objectives and behavioural determinants, (3) selecting theory-based behaviour change methods, (4) organising the programme and (5) implementing and evaluating intervention (see Fig. 1). This paper focusses on steps 1–4. Implementation and evaluation are described elsewhere [16].

### Stage 1: needs assessment

The first stage of IM involves carrying out a needs assessment to establish the extent of the health problem and identify determinants associated with the problem behaviour. This needs assessment was informed by a broad review of the literature, using search terms around ‘tamoxifen’ ‘endocrine therapy’, ‘adherence’ and ‘persistence’, with reference to several systematic reviews [17–19]. In addition to this, the authors carried out a qualitative study and a longitudinal observational study which used the CSM and TPB as a framework to understand factors associated with non-adherence [20, 21].

### Stage 2: identify intervention objectives

The next step of the IM process involves identifying the desired outcomes of the intervention. Specific objectives related to improving adherence were identified, informed by the needs assessment and based on theory-based constructs outlined in the CSM and TPB. These objectives were broken down to determine the exact steps to improve adherence. This stage of the process allows researchers to be very clear and precise about what the intervention objectives are, and what determinants need to change in order to achieve these objectives.

### Stage 3: identify theory-based methods and practical strategies

The third stage involves identifying theory-based health behaviour change methods and techniques to map onto the determinants identified in the needs assessment. This was achieved through a thorough literature review, searching for studies which had modified the determinants identified in the intervention objectives.

### Stage 4: develop intervention

The previous stages of the IM process identified a series of determinants which formed the intervention targets, and a number of behavioural strategies which could modify these determinants. This stage of IM involves combining these behavioural strategies in order to develop the intervention programme. The development was an iterative process and was informed by feedback from the research team and clinicians. Two nurse specialists reviewed the content of the intervention. In addition, as per recent guidance, patient representatives were involved in the development of the intervention from the very beginning [13]. Interviews with three patient representatives took place before the intervention was developed in order to discuss the format and scope of the intervention. These patient representatives were recruited from a database from a previous study. The intervention materials were then reviewed by nine patient representatives via email. These representatives were recruited through advertisements on Facebook groups and through a college-wide circular. The patient representatives were all breast cancer survivors prescribed tamoxifen for primary breast cancer and were aged between 36 and 67.

## Results

### Stage 1: needs assessment

The needs assessment highlighted the extent of non-adherence in this population, with adherence rates ranging from 47 to 97% and falling over time [17, 18]. Unintentional non-adherence was reported much more frequently than intentional non-adherence and was associated with unique determinants [9, 21, 22]. Key barriers and facilitators of tamoxifen non-adherence identified by the needs assessment are shown in Table 1. More positive medication beliefs, such as higher necessity beliefs and lower concerns, have consistently been associated with improved HT adherence and persistence [9, 21, 23–25]. Recent research has also highlighted the utility of illness perceptions, defined as the beliefs that patients hold about their illness, in understanding non-adherence to tamoxifen. For example, women who reported via the Illness Perceptions Questionnaire for Breast Cancer Survivors (IPQ-BCS; [26]) higher consequences of taking tamoxifen and who thought that their likelihood of cancer recurrence was low were more likely to be non-adherent. Additionally, women who believed that psychological factors such as stress or emotional state are important in causing a recurrence were more likely to be non-adherent, whereas women who believed that health behaviours such as diet and exercise are important in causing a recurrence were less likely to be non-adherent [9, 21]. From the Theory of Planned Behaviour, perceived behavioural control, which refers to the amount of control a patient feels they have over taking tamoxifen, and positive attitudes towards tamoxifen have been shown to be associated with increased adherence [9, 21, 22].

**Table 1:** Key determinants of non-adherence (and non-persistence) identified by the needs assessment

	Barrier to tamoxifen adherence	Facilitator of tamoxifen adherence
Medication beliefs	Concerns about medication [9, 21, 23]^a^ Specific concerns (e.g. risk of endometrial cancer)	Necessity beliefs [9, 23]^a^ [22, 24, 25]
Illness perceptions	Tamoxifen consequences [9]^a^ Causal beliefs (psychological attributions) [9, 21]^a^	Stronger beliefs in risk of recurrence [9]^a^ Causal beliefs (health behaviours) [9]^a^
Theory of planned behaviour	-	Coherence [21] Positive attitude towards tamoxifen [21] Self-efficacy for taking medication [22]^b^ Perceived behavioural control [9]^a^ [21]
Side effects	Number/intensity of side effect experience (e.g. fatigue, vaginal dryness) [22, 23]^a^ [4, 21]	-
Social support	-	Perceived good social support [21, 27] Perceived self-efficacy in patient/doctor relationship [28]
Knowledge	Lack of information about treatment [22, 27, 29]	Information about treatment is understandable [27]
Distress	High levels of distress [21]	-

^a^Indicates that a factor may be associated with intentional non-adherence

^b^Indicates that a factor may be associated with unintentional non-adherence. References are not exhaustive

As tamoxifen lowers circulating oestrogen levels, it is associated with menopausal related side effects, such as hot flushes, night sweats and loss of libido. These side effects can have a significant impact on quality of life [30] and are often identified as drivers of non-adherence [4, 18, 19]. Studies suggest that women often feel unprepared for the side effects and that they are given little support in coping with them [20, 29]. Whilst the relationship between side effects and adherence is not always consistent, qualitative research has shown that many women stop taking tamoxifen because of their side effects [20].

A range of studies have also identified lack of information as a barrier to tamoxifen adherence, with women who feel more informed about treatment being more likely to adhere [22, 27, 29]. This is an important issue, as research has shown that many women report a lack of information provision or knowledge about tamoxifen [20, 29]. Finally, low levels of social support and high levels of distress have also been identified as facilitators of non-adherence [21, 27].

### Stage 2: identify intervention objectives

The needs assessment identified that the intervention should aim to address both intentional and unintentional non-adherence, with a focus on unintentional non-adherence as this was reported more frequently. A number of determinants of adherence which may need to be modified in order to see improvements in adherence were also identified (see Table 1).

Modifying the following determinants formed part of the intervention objectives:

- Increasing knowledge of, and beliefs about the necessity of, tamoxifen
- Reducing concerns about tamoxifen
- Reducing impact of side effects
- Increasing social support
- Reducing likelihood patient will forget tamoxifen
- Supporting development of accurate illness perceptions

**Fig. 2:**
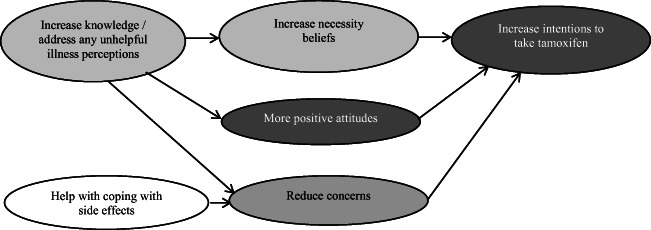
Theoretical interpretation of the inter-connectivity between key variables. Dark grey boxes indicate constructs based on the TPB and light grey boxes indicate constructs based on the CSM

Some of the factors in Table 1 overlap or map onto each other and can therefore be targeted together (Fig. 2). For example, increasing knowledge should help to increase necessity beliefs and create more positive attitudes towards tamoxifen, which should also increase intentions to take tamoxifen [31, 32]. Encouraging more accurate risk of recurrence perceptions may also increase necessity beliefs. Furthermore, women’s concerns focus on side effects, and therefore, increasing women’s confidence in managing these side effects may help to reduce their concerns, resulting in a more favourable cost-benefit analysis.

### Stage 3: identify theory-based methods and practical strategies

Many studies have suggested that improving the extent to which women feel informed about treatment should improve HT adherence rates [33, 34]. Providing information on the clinical benefits of tamoxifen is particularly important in a preventive medication like tamoxifen where the benefits are hidden, and where no reduction in symptoms can be attributed to medication taking [35]. This means that there is no overt positive reinforcement for the patient to continue taking the medication. However, previous interventions to improve HT adherence have focussed mainly on providing information and have been shown to be largely ineffective at improving adherence [8]. It has been suggested that the efficacy of interventions like these could be improved if combined with behavioural interventions or strategies to address medication beliefs [8]. Therefore, the current intervention included a range of different strategies and activities alongside information provision. The information presented was multi-modal, with written, visual and audio information, in order to cater to different learning needs. Quotes and videos from other women were incorporated throughout the intervention to increase engagement. Additionally, a range of evidence-based theories and strategies were used to increase the likelihood of behaviour change, including elements from cognitive behaviour therapy (CBT) such as goal setting and cognitive reframing. These strategies are shown in Table 2 and are discussed below in relation to each of the key determinants associated with non-adherence.

**Table 2:** Strategies for modifying key determinants associated with non-adherence

Key determinants	General method for addressing determinant	Specific strategies/techniques	Intervention section
Medication beliefs	Increase necessity beliefs	Providing information on why tamoxifen is necessary, how it works and what happens if doses are missed. Visual information (diagrams) to demonstrate the mode of action, quotes/videos for social comparison.	1, 2
	Address concerns	Providing information on common concerns. Activity to address concerns and challenge any misconceptions of medication.	1, 2, 3
Illness perceptions	Challenge unhelpful beliefs about illness	Providing information on how tamoxifen works, how effective is. Activity to challenge inaccurate beliefs.	1, 2
	Reduce tamoxifen consequences	Providing information on how to manage side effects. Goal setting activity, videos and quotes for social comparison.	3
Theory of planned behaviour constructs	Increase intentions, develop more positive attitudes	Providing information on how tamoxifen works, its effectiveness, consequences of not taking medication. Addressing concerns associated with medication.	1, 2
	Bridge gap between intentions and behaviour	Implementation intentions activity, goal setting/action planning, evaluation of goal setting.	2
	Improve perceived behavioural control	Tips for taking tamoxifen, social comparison, goal setting/action planning.	2
Side effects	Develop coping skills and enhance self-efficacy	Providing information on practical tips and coping strategies for common side effects. Psychoeducation on why side effects may occur. Symptom monitoring, quotes and videos for social comparison, enhance confidence for dealing with symptoms.	3
	Set goals for managing symptoms	Formulate SMART goals, implement goals, evaluate goal setting.	3
	Use CBT strategies to help reduce impact of HFNS	Psychoeducation on the physiology of HFNS, identify potential triggers, challenge negative thoughts about HFNS, develop more helpful responses, paced/diaphragmatic breathing.	3
	Use CBT strategies to help reduce fatigue	Psychoeducation on consequences of erratic patterns of rest and activity/over-activity, importance of establishing good sleep patterns and a balance of rest and activity, challenge unhelpful thoughts and behaviours which perpetuate fatigue, provision of information on sleep hygiene.	3
Social support	Increase perceived social support, encourage women to seek support	Providing information on the importance of asking for help, quotes and videos for social comparison. Provide resources for seeking social support elsewhere and for seeking professional help.	4
Knowledge	Information provision	Psychoeducation, visual information, signposting to further information, evaluation of knowledge.	1, 2
Forgetting	Strategies to help remember to take tamoxifen, increase motivation to remember	Practical tips, social comparison, implementation intentions, information on consequences of non-adherence.	2

Note: *CBT* cognitive behaviour therapy, *HFNS* hot flushes/night sweats, *SMART* specific, measureable, achievable, relevant, time-limited

Beliefs about tamoxifen, and to a lesser extent, beliefs about breast cancer, were identified as potential determinants of non-adherence in the needs assessment. As shown in Table 2, effective strategies to modify these beliefs include providing information to modify inaccurate perceptions; challenging unhelpful beliefs; discussing behaviour change methods and; encouraging patients to generate responses to overcome their concerns [36–38]. Several studies have seen significant improvements in adherence rates following modification of illness and treatment perceptions [38]. Studies have also used diagrams or demonstrations of a medication’s mode of action in order to modify treatment beliefs [31]. These help to turn intangible information into more concrete representations and can improve adherence rates [39].

Constructs from the TPB were identified as key determinants of non-adherence in Table 1 and techniques based on these constructs were integrated into the intervention (Table 2). Interventions based on this theory have shown success at improving adherence [40]. For example, several studies have successfully used implementation intentions, which involve pairing a critical cue (i.e., morning coffee) with the goal-directed response (i.e., taking medication), thus establishing habit and removing the cognitive burden for patients to remember their medication [41].

Another key determinant of non-adherence is side effects. Research suggests that informing women which side effects to expect and helping them to manage side effects should improve adherence rates [42]. Several studies have shown that techniques such as providing clinical information on side effects, sharing experiences to empower patients, enhancing problem solving skills, cognitive reframing, and relaxation can improve side effect management and quality of life [43, 44]. CBT techniques have also shown success at improving symptom management, in patients with fatigue or with hot flushes and night sweats [44].

### Stage 4: develop intervention

Once the theory-based methods and practical strategies were identified (Table 2), the format of the intervention programme was decided on. In this case, a self-management intervention was chosen, where patients would work their way through a paper booklet in their own time. It was felt this would have the widest reach, especially in light of Open Access Follow Up being implemented for breast cancer survivors in the UK, meaning women have limited contact with their breast care team following primary treatment. A self-management intervention has the potential to be disseminated via a broad range of channels such as patient advisory groups, GPs and Breast Cancer specialist services, without requiring extensive resources to support implementation.

Patient representatives provided feedback on the format and scope of the intervention, agreeing that a self-management booklet would be helpful and appropriate. The overall feedback on the intervention materials was very positive, but some small changes were made based on patient feedback (Table 3). Constructive suggestions included reducing some repetitive information and adding additional infographics and clinical information. Negative feedback tended to be focussed more around pragmatic issues, such as the use of the colour pink or the ordering of some of the information.

**Table 3:** Feedback on the intervention materials from patient representatives

Quotes from patient representatives on the intervention materials	
“Very excited about this booklet as it is desperately needed”	
“I think it’s great!! It’s easy to read, very informative, more so than when I was originally diagnosed. The exercises are a great idea”	
“This all looks great to me, really informative and I can't think of anything that you haven't covered. I wish I had something like this to read when I started tamoxifen!”	
“The diagrams are brilliant as they really help explain everything”	
“The information given is very detailed and useful. I especially like (and can identify with) the comments given by ladies taking tamoxifen.”	
“Wish I’d had this booklet from the beginning!”	
“All I can say is wow. I have read through all of it and have made mental notes to myself on how I will cope for the next 5 years. I can’t see anything negative to report back on”	
“I'm personally not keen on the use of the colour pink in breast cancer resources. It has become a way of “branding” breast cancer and I think this pinkification relates to rather stereotyped ideas of femininity.”	
“I don't think you do anything like enough on the joint pain. Honestly needs more! You also list it as common issue in one part, but it’s not on first page. I struggle most mornings and have sore hips/ knees.”	

### Programme content

**Fig. 3:**
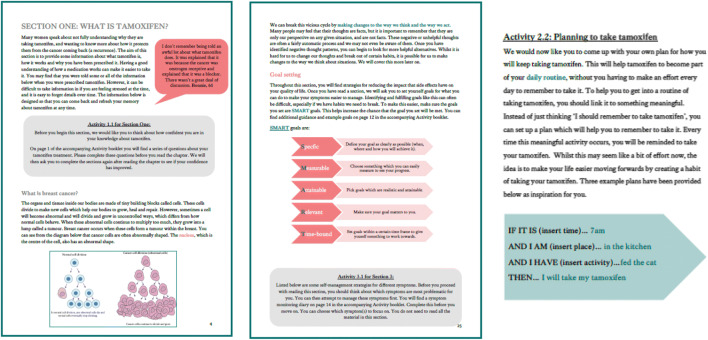
Intervention content

The intervention booklet was split into four sections. Each of these sections included information, activities and quotes and access to videos from other breast cancer survivors. All patients read the first section (*What is tamoxifen?*), and then were directed to complete a questionnaire designed to highlight their particular areas of need. Patients then chose which of the following sections to focus on based on their tailored responses. Example screenshots from the intervention booklet are shown in Fig. 3 and Online Resource 1.

#### Section 1

The first section of the booklet provides information on what tamoxifen is and how it works. To increase comprehension and in an attempt to modify medication beliefs, this section also includes diagrams to demonstrate how cancer cells can be stimulated and how tamoxifen can help to reduce this risk. Glossaries of key terms and signposting to sources of information are also included.

#### Section 2

Section 2 focusses on how to take tamoxifen and addresses both forgetting to take tamoxifen (unintentional non-adherence) and deliberately skipping doses (intentional non-adherence). The aim throughout this section is to educate patients on the importance of taking tamoxifen as prescribed, whilst normalising forgetting and understanding that for some women, non-adherence or non-persistence may be the best solution based on their personalised risk of cancer recurrence and their quality of life. Some information from Section 1 is reiterated here, to explain what happens when doses are missed. Evidence suggests that some women who are non-adherent still feel they are appreciating the full clinical benefits of treatment [20]. Therefore, it was important to address this perception and provide more accurate information. To address unintentional non-adherence, women are given tips from other patients on remembering to take tamoxifen and how to improve planning. They are then encouraged to complete an implementation intention activity, where they pair the behaviour of taking tamoxifen to a key activity in their day, such as a morning cup of coffee. Participants write their plan down in the template provided, visualise it and repeat it until they can recite it from memory. To address intentional non-adherence, information is provided to debunk five common concerns about tamoxifen. Women are then asked to list their personal concerns and then use problem solving to provide a response to overcome this concern.

#### Section 3

The third section focuses on how to manage common side effects. Patients are introduced to the link between thoughts, feelings and behaviours, before completing a symptom monitoring diary to inform which symptoms to focus on. Information is then provided for six common side effects: hot flushes and night sweats (HFNS); vaginal dryness/itchiness/discharge; tiredness/fatigue and insomnia; changes in mood; weight gain and; joint pain. Each section includes information on why the side effect may occur and tips for symptom management. After reading each section, patients are referred to an activity where they set a SMART goal to manage their symptoms. After spending 2 weeks implementing this goal, patients review their progress and make amendments to their goal if necessary. Examples are provided throughout to support women in trying new techniques.

The section on HFNS was informed by the successful CBT treatment for HFNS [43]. The sections on tiredness, fatigue and insomnia also utilise CBT techniques by reiterating the link between thoughts, feeling and behaviours and providing fatigue management techniques such as balancing rest and activity, keeping a fatigue diary and practising good sleep hygiene. The remaining sections provide a range of different tips for managing the symptoms, as well as quotes from breast cancer survivors and resources for more information or support. The aim of the section was not to remove the side-effects, as this is unlikely to be successful, but to improve women’s confidence in coping with them and to reduce their impact on quality of life.

#### Section 4

The final section focusses on increasing access to social support, which was identified as a potential determinant of non-adherence. The section focusses on normalising the need for additional support and encouraging women to seek support from a range of sources. Resources are provided for finding helplines, support centres and face-to-face or online support groups. This section also addresses communication with healthcare professionals and encourages women to discuss their concerns with their healthcare team.

## Disc**u**ssion and conclusion

### Discussion

This paper describes the development of an intervention to improve adherence in breast cancer survivors prescribed tamoxifen. The intervention was developed following an iterative process involving feedback from researchers, clinicians and patients. The intervention aims to support breast cancer survivors with their tamoxifen treatment, by helping them to manage their side effects, to remember to take the medication and to gain more understanding of how tamoxifen works. Preliminary feedback from patient representatives was positive, with women stating it was helpful and wishing it was available for them when they started treatment. The intervention has now been tested in a small pilot study with 27 women who completed questionnaires pre and post the interventions materials. Uptake and retention rates showed that the intervention was acceptable and feasible, and paired sample *t* tests showed small to moderate improvements in adherence and a range of secondary outcomes, such as distress and quality of life [16].

This article meets the need for calls for more thorough descriptions of interventions, which are currently lacking [45], and which discuss not just what is included in the intervention, but why it is included. This was achieved in the current paper by following the Intervention Mapping (IM) framework, which is a thorough and transparent method for developing interventions [14], allowing for very clear descriptions of how the intervention was developed, and showing the theoretical and empirical underpinnings of strategies used in the intervention. As well as following the IM framework, the intervention was based on two models of health behaviour (The Common Sense Model and the Theory of Planned Behaviour). These models were chosen as evidence has supported their use in understanding and predicting HT non-adherence [9, 21]. Previous interventions to improve adherence are often not theoretically grounded or evidence based, which may contribute to the lack of efficacy seen across interventions [10]. The development was also in line with the MRC guidance for complex interventions, which states that interventions should be developed systematically based on the best available evidence and with theoretical understanding of the process of change [13]. Following guidelines and frameworks such as these increase the likelihood that the intervention will be effective [7].

Whilst the intervention was developed in a thorough and rigorous process, there are some limitations with the development process. The patient representatives in this study may have experienced some social desirability bias to please researchers, but this was overcome by collecting feedback over email and encouraging patients to be open and honest with their feedback. Furthermore, the patient representatives had no prior relationship with the research team. Strengths of the process include the breadth of evidence considered in the needs assessment, the use of evidence-based strategies for behaviour change and the input from patients and from a multidisciplinary team.

### Implications

Following the Intervention Mapping guidelines allowed this intervention to be developed in a transparent and systematic manner with a sound empirical and theoretical rationale. This process has likely enhanced the quality of the intervention and improved the likelihood it will be efficacious. The intervention has now been piloted in a small feasibility study which has shown small to moderate sized positive effects in adherence and a range of secondary outcomes including satisfaction with information about medication, illness and treatment beliefs and self-efficacy in managing symptoms [16]. Due to the very large numbers of women with oestrogen receptor positive breast cancer being prescribed tamoxifen, this intervention has the potential to be rolled out widely, benefitting many women at a low cost. However, larger-scale trials are needed to establish the efficacy of the materials.

## Supplementary information

ESM 1 (DOCX 664 kb)

## Data Availability

Not applicable.
