# Supplementary figures and images for: Development of a self-management intervention to improve tamoxifen adherence in breast cancer survivors using an Intervention Mapping framework
Source: Support Care Cancer. 2020 Oct 29;29(6):3329–38. doi: 10.1007/s00520-020-05850-x (PMC8062369; doi:10.1007/s00520-020-05850-x)

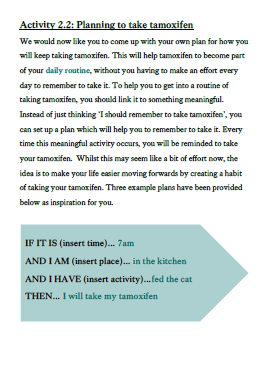

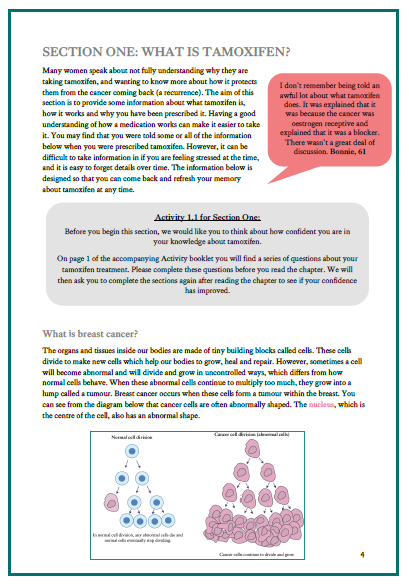

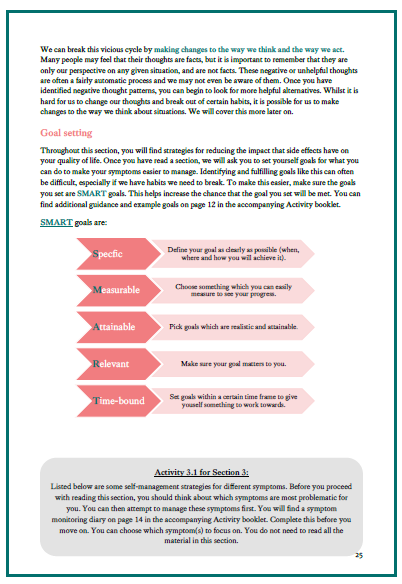


Online Resource 1: Example screenshot of intervention content

Supplement: Supplementary file 1 — (DOCX 664 kb) [file 520_2020_5850_MOESM1_ESM.docx]
